# Effects of low-frequency burst stimulation of the motor thalamus on cortical neural co-firing

**DOI:** 10.1016/j.brs.2025.103018

**Published:** 2026-01-03

**Authors:** Kyungsoo Kim, Giri P. Krishnan, JaeYeon Kim, Sergio Arroyo, Maxim Bazhenov, Karunesh Ganguly

**Affiliations:** a Department of Neurology, University of California, San Francisco, CA, United States; b San Francisco VA Healthcare System, University of California, San Francisco, CA, United States; c School of Computational Science and Engineering, Georgia Institute of Technology, Atlanta, GA, United States; d Department of Medicine, University of California, San Diego, CA, United States

**Keywords:** Low-frequency burst stimulation, Motor thalamus, Neuromodulation, Co-firing modulation, Thalamocortical circuit

## Abstract

**Background:**

Electrical stimulation targeting the motor thalamus (Mthal) represents an emerging strategy for regulating motor cortical activity patterns, however the fundamental mechanisms and optimal stimulation configurations are not well established.

**Objective:**

To characterize how burst Mthal stimulation modulates cortical activity and to identify stimulation parameters that maximize cortical co-firing via thalamocortical rebound mechanisms.

**Methods:**

We conducted acute electrophysiological experiments in anesthetized rats using Mthal stimulation with systematically manipulated burst stimulation parameters (i.e., number of pulses and pulse width) in the low-frequency band (1–8 Hz) modulation cycle. Cortical responses across large areas covering primary and secondary motor cortex were recorded. Additionally, intracortical recordings were performed for detailed spike activity monitoring during stimulation. Cortical activity patterns in local field potentials and spike activity were analyzed to quantify the effects of Mthal stimulation on motor cortex. Computational thalamocortical circuit models were employed to explore the mechanistic basis of frequency-selective modulation effects.

**Results:**

Burst Mthal stimulation elicited tunable cortical excitation, with optimal modulation achieved at 3–4 Hz. This preferred frequency corresponded to the natural rebound timing of thalamocortical (TC) cells and generated maximal cortical co-firing and synchronization. Higher stimulation frequencies (e.g., 8 Hz) resulted in suppressed and diminished responses. Computational modeling further validated that the cellular mechanism of the TC cell rebound excitation leads to frequency-dependent cortical modulation.

**Conclusion:**

Burst Mthal stimulation demonstrates frequency-selective modulation of cortical network excitability, with 3–4 Hz protocols providing optimal enhancement of synchronized activity through exploitation of thalamocortical rebound dynamics. These results establish motor thalamic stimulation as a promising and efficient methodology for precise cortical network control and offer mechanistic foundations for developing therapeutic neuromodulation interventions aimed at motor system rehabilitation.

## 1. Introduction

Low-frequency cortical dynamics (1–4 Hz) play a crucial role in task preparation and execution [1–6]. This low-frequency activity is closely linked to task-related population co-firing patterns, reflecting the underlying network connectivity among cortical and subcortical regions [7,8]. Disruptions to this network can lead to diminished or enhanced low-frequency dynamics [9], suggesting an important role in sustaining motor functions.

Previous studies have demonstrated that low-frequency modulation (e.g., <4 Hz) through direct cortical stimulation can enhance cortical activity and improve motor function following stroke [7,9–11]. Specifically, diminished cortical activity was restored through epidural low-frequency current stimulation, which led to increased ensemble co-firing and improved task performance. Here, we aimed to evaluate how deep brain stimulation (DBS), which is widely used clinically and can target smaller areas within deeper brain structures [12,13], can be used to modulate motor networks in a manner that mimics low frequency stimulation. The effects of DBS are highly dependent on the target region and stimulation parameters, particularly frequency, which can either suppress or promote neural activity [14]. Continuous frequencies below 80 Hz tend to excite, while those above 100 Hz inhibit somatic activity in the targeted region [15–17]. We specifically aim to test how ‘burst-modulation’ of DBS stimulation (i.e., patterned stimulation as opposed to continuous stimulation) might modulate cortical dynamics and mimic low-frequency modulation.

The motor thalamus (Mthal) projects to extensive regions of the motor cortex [18–22], making it a promising target for network-level modulation that may be more effective than direct cortical stimulation. Thalamic input has been shown to strongly influence cortical rhythmicity and population co-firing [23,24], underscoring the potential of thalamic stimulation for state-dependent modulation of cortical dynamics. Moreover, thalamic stimulation can dynamically shape cortical trajectories in real time, influencing both behavioral outcomes and circuit-level flexibility [25,26]. These findings collectively suggest that the thalamus is a potent site for behaviorally relevant modulation of cortical dynamics.

We aim to explore the modulation of activity across large areas of the motor cortex. However, complex cellular and network mechanisms influence the response and must be considered to identify effective stimulation parameters. Thalamic cells broadcast oscillatory signals, facilitated by specialized channels such as hyperpolarization-activated cyclic nucleotide-gated (HCN) channels [27] and low-voltage-activated (LVA) calcium channels [28], alongside recurrent feedback loops with the reticular nucleus (RE) [29]. While existing stimulation approaches have aimed to enhance cortical activity at a variety of frequency ranges 24, there is a lack of detailed exploration regarding how stimulation interacts with the intrinsic excitability of thalamocortical circuits. We hypothesize that Mthal stimulation’s impact on cortical activity arises from modulation of the oscillatory mechanisms inherent in thalamic neurons.

To test the feasibility of utilizing Mthal stimulation to enhance low-frequency cortical dynamics, we have systematically characterized and optimized Mthal stimulation to achieve wide-spread modulation of cortical activity. Our approach utilizes an anesthetized rodent model for stimulation parameter optimization and is complemented by in-silico analyses to suggest putative mechanisms underlying Mthal and cortical responses to stimulation.

## 2. Methods

### 2.1. Surgery and electrode placement

**Fig. 1.**
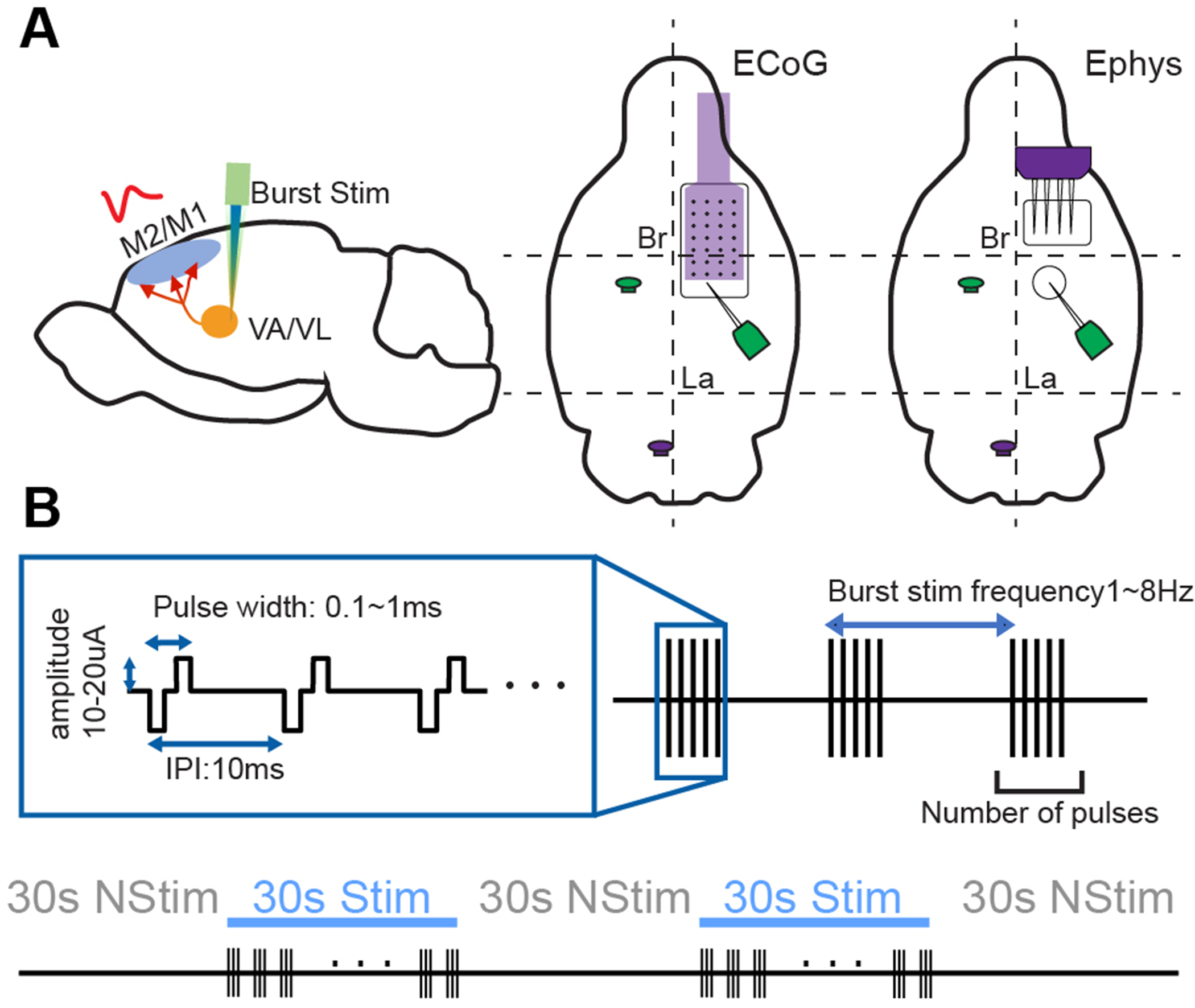
Experimental overview. (A) Electrode placements for stimulation and recording shown in sagittal (left) and horizontal (middle, right) brain sections. Burst stimulation was delivered to the motor thalamus (VA/VL), and electrocorticography (ECoG, middle) and intra-cortical electrophysiology (ephys, right) was recorded from the motor cortex (M1/M2). (B) Burst stimulation protocol consisting of biphasic square-wave pulses with variable pulse width (0.1–1 ms), amplitude (10–20 μA, see the detailed amplitude for each animal in Table 1), and inter-pulse interval (10 ms). Bursts were delivered at frequencies ranging from 1 to 8Hz with varying numbers of pulses per burst. Each stimulation epoch lasted 30s, followed by 30s of no stimulation (NS), repeated in alternating cycles.

Experiments were approved by the Institutional Animal Care and Use Committee at the San Francisco VA Medical Center (Protocol #25-001). All surgical procedures were conducted under sterile conditions in accordance with institutional animal care guidelines. Adult Long-Evans rats (Male, >10 weeks old, >400g, n = 4 for electrocorticography, ECoG, and n = 4 for multi-array electrophysiology) underwent acute anesthetized recordings. Under 2–4 % isoflurane, surgery involved cleaning and exposure of the skull. Craniotomy windows were made for electrode placement; one large cranial window for both ECoG electrode (AP: 2 to −2.5 mm, ML:1–3 mm) and Mthal stimulation electrode (AP: −1.6 to 2.6 mm, ML: 1.5–2.5 mm) as shown in Fig. 1A. For electrophysiological recordings from layer2/3 to layer 5, two separated cranial windows were made for electrophysiological recording (AP: 0.7–2.7 mm ML: 1–3 mm) and Mthal stimulation electrode as shown in Fig. 1A. Note that All stereotactic coordinates were referenced to bregma, and targeting of the VA/VL thalamic region was guided using the rat brain atlas from labs.gaidi.ca. Skull screws were placed ~1 mm posterior than lambda and ipsilateral area of Mthal craniotomy (AP: −2.1 mm, ML: 2 mm) for joint ground (GND) and reference (REF) wire of recording electrode and stimulation electrode respectively. We separated recording and stimulation GND/REF pair to prevent unexpected current flow between them. ECoG electrode (AP: 1.1 to −1mm, ML:1–2 mm, Neuronexus, E32-300-20-50) and four shank multi array electrode (AP: 1 mm ML: 1–3 mm, DV: 0.5–1.5 mm, Neuronexus, A4x8-5mm-200-400-177) were placed at primary (M1) and secondary motor (M2) area. One shank micro electrode was implanted to Mthal region targeting the ventral anterior (VA) and ventral lateral (VL) nuclei (AP: −2.1 mm, ML: 2 mm, DV: ~6 mm, Neuronexus, A1x16-10mm-100-703). During the early phase of the study, histological verification was performed to confirm electrode placement relative to VA/VL boundaries. We dipped the shank into DiI (Dil Stain, ThermoFisher Scientific, 2.5 mg/mL) five times, allowing approximately 3 min of air-drying between each dipping; this allowed us to assess the localization of electrodes. Brains were subsequently collected and sectioned for histological verification, and these images confirmed that the stimulation site was situated within the VA/VL thalamic region, consistent with the intended targeting (Fig. S1A–B). In later experiments, functional localization was refined by monitoring the characteristic cortical response patterns evoked by Mthal stimulation (i.e., evoked potential and aligned spike co-firing activity), with only minor vertical adjustments (<0.2 mm) required. These evoked responses provided a reliable and reproducible physiological marker for accurate targeting. In many cases, optimal electrode positioning was achieved with stimulation amplitudes of 10–15 μA (Table 1), and differences in the optimal amplitude likely reflected small variations in electrode placement across experiments.

Anesthesia was induced with an intraperitoneal injection of ketamine (80 mg/kg) and xylazine (10 mg/kg). More than 1 h prior to Mthal stimulation and cortical recording, isoflurane anesthesia was discontinued, and animals were transitioned to ketamine/xylazine anesthesia. Anesthetic depth was monitored throughout the procedure by assessing the pedal withdrawal (toe pinch) reflex. At approximately 50 min after induction, the pedal withdrawal reflex was re-evaluated, and supplemental doses (equivalent to 50 % of the initial ketamine/xylazine dose) were administered intraperitoneally every 30 min as needed to maintain a surgical plane of anesthesia. All pinch testing and supplemental injections were performed during non-recording conditions. Body temperature was maintained using a thermostatically controlled heating pad throughout the procedure.

### 2.2. Histological imaging

Rat brains were collected following transcardial perfusion and fixed in 4 % paraformaldehyde (PFA) in PBS for 1 day at 4 °C. Cryopreservation was in 30 % sucrose in PBS at 4 °C until tissue specimens sank to the bottom of the vial. The brain was frozen in an optimal cutting temperature (OCT) compound and then was cut at ~40 μm on a cryostat and mounted on glass slides for experimental analysis. The slide was stained with DAPI solution for 30 s at room temperature and mounted with ProLong Gold Antifade Mountant solution (Thermo Fisher Scientific, cat# P36934). Tiled images of entire slides were acquired on a Leica Widefield at a 10X objective (NA 0.45), and DiI fluorescence was detected using the Cy3 excitation channel.

### 2.3. Motor thalamus stimulation

Motor thalamic stimulation was delivered using a Tucker-Davis Technologies (TDT) IZ2 stimulator, and stimulation parameters (amplitude, biphasic pulse design, pulse width, number of pulses per burst, and stimulation frequency) were controlled using the Synapse software interface. A biphasic pulse which has symmetric charge balance waveform was formed with amplitude and pulse width. In addition, we pursue to use burst stimulation which can bring larger stimulation effect using multiple pulses. We maintained the stimulation amplitude within 10–20 μA to limit the effective stimulation area and increased the charge by extending the pulse width (i.e., 0.1–1 ms 40 % of cathodic square, 20 % of delay and 40 % of anodic square duration). The burst stimulation had an inter-pulse interval of 10 ms (100Hz). In the intracortical micro-array experiments (n = 4), pulse width ranged from 0.1 to 1.0 ms in 0.1 ms increments, and burst pulse number was set to 1, 3, 5, or 8 pulses. Stimulation frequency ranged from 1 to 8 Hz in 1 Hz steps. In the ECoG experiments, a single stimulation condition was applied (1 Hz, 5 pulses, PW = 1 ms, n = 4). Each frequency condition consisted of repeated 30-s stimulation periods followed by 30-s non-stimulation intervals, for a total duration of ~10 min per each frequency condition. Short inter-condition breaks (<5 min) were used to assess anesthesia depth (pedal withdrawal reflex and respiration) and maintain a stable physiological state (Fig. 1B).

### 2.4. Electrophysiology recording and pre-processing

ECoG and intracortical local field potential (LFP) signals were recorded using the Synapse system (Tucker-Davis Technologies) at a sampling rate of 1024 Hz. Spikes were recorded with 24 kHz sampling rate and then band-pass filtered (1k-10 kHz). Spike detection was performed with thresholded at 4 standard deviations, and sorted using the Wave-clus algorithm in MATLAB [30]. To minimize stimulus-related artifacts, the stimulation and recording grounds were electrically separated and only indirectly coupled through brain tissue. Under this configuration, stimulation artifacts were present but remained small in magnitude. LFP signals were processed using band-pass filtering and a median moving filter to suppress residual stimulation transients. Spike waveforms were inspected, and non-physiological waveforms (e.g., double-peak shapes) were removed manually to ensure that only stable and biologically plausible spike waveforms were retained (Fig. S1C–D). The resulting datasets were used for all subsequent analyses.

Because higher stimulation frequencies yield more stimulus events within a 30-s stimulation period, the number of trials per condition was normalized by selecting the 25 stimulus-locked events (corresponding to each integer second for 25s after stimulation onset) for analysis across all conditions. This procedure ensured consistent trial counts and comparable signal-to-noise ratios across stimulation frequencies. Non-stimulation baseline trials were sampled once every second from the 25-s non-stim period immediately preceding each stimulation block (total of 25 trials), and were aligned and analyzed using the same trial-averaging procedures as the stimulation-locked data.

### 2.5. Neural data analysis

For ECoG, We utilized LFP to estimate cortical activity change after stimulation, we measured the first upstate (N1, first negative peak) and the first downstate (P1, first positive peak). Modulated amplitude in LFP was calculated by the peak amplitude difference between N1 and P1.

Multi-channel LFP recordings from the intracortical multi-array were used to analyze the responses to Mthal stimulation. Peak-to-peak analysis was performed in the same manner as in the ECoG analysis. Principal component analysis (PCA) was applied to identify the dominant stimulation-evoked activity patterns. The first two principal components, which accounted for the largest proportion of variance, were used to obtain a low-dimensional representation of the cortical response dynamics across trials and stimulation conditions. This approach reduced noise and minimized contributions from stimulation-unrelated activity, allowing consistent comparison of response waveform structure. For time-frequency analysis, LFPs were low-pass filtered at 10 Hz to avoid phase distortion and then downsampled to have 20Hz sampling rate. Spectrograms were computed by spectrogram function in MATLAB with a 50-sample Hamming window, 80 percent overlap window, and 128-point FFT.

Intracortical spike activity was utilized for cortical cell activity. The cells, that were spike sorted, were used for quantitative analysis. We classified the spike response into two groups, initial response (<100 ms post stimulation) and rebound (>100 ms after post stimulation silence). Trial averaged spike delay was calculated by averaging spike response time after stimulation.

The averaged correlation coefficient was obtained by computing the Pearson correlation for each possible pair of cell activities, excluding self-self pairings, and then averaging these values across trials and cell pairs as follows,

$$Aver.\;Corr.\;Coef=\frac{1}{K.N}\sum_{k=1}^{K}\sum_{i=1}^{I}\sum_{j=1,j\neq i}^{J}r_{c_{i},cj}^{\left(k\right)}$$

Where $r_{c_{i},cj}^{\left(k\right)}$ denotes the Pearson correlation coefficients between the *i*th cell *c_i_*, and *j*th cell *c_j_* during trial *k*.

For cell modulation, the spike count in 60 ms time bin before and after stimulation was calculated and used for two tailed *t*-test. the cells showing significant difference (p < 0.05) was considered as modulated cells. Modulated cell ratio is calculated based on the number of modulated cells/total number of cells. To assess cell activity coupling, we calculated the correlation coefficients across all possible single-cell pairs. The neural data analysis was performed with customized code in MATLAB.Gaussian Process Factor Analysis (GPFA) was used to extract low-dimensional latent trajectories from high-dimensional spike trains. Spike co-firing during different Mthal stimulation frequencies was quantified based on the activity of all recorded cells. Spiking activity was binned with 50 ms window before applying GPFA to identify population-level co-firing dynamics over time by DataHigh library in MATLAB [31].

### 2.6. Thalamocortical network simulation for Mthal stimulation

To investigate the detailed stimulation effect on thalamocortical network (TC-Net), we utilized a computational model of thalamocortical networks. The TC-Net model is based on the thalamocortical network model [32], composed of 500 pyramidal (PY), 100 inhibitory cortical neurons (IN) with 100 thalamocortical (TC) cells and 100 reticular nucleus (RE). The network is connected by excitatory and inhibitory synaptic connections (i.e., AMPA, MNDA, GABAa and GABAb). Key intrinsic properties were of each neuronal subtype to reflect biologically plausible dynamics. The TC cells included various ion channels, including HCN and LVA (low-threshold Ca^2+^ current) calcium channels (h- and t-channel respectively), which are involved in rebound and oscillatory activity. Burst stimulation was delivered via a current clamp applied to TC neurons. The stimulation protocol involved 3-pulse bursts, each pulse with a duration of 10 ms with 50 % of duty cycle and an amplitude of 12 μA/cm^2^ 20 % of TC neurons received full stimulus intensity (normalized weight = 1), while the remaining 80 % received linearly scaled weights between 0 and 1 (Fig. 8A bottom).

Stimulation was applied over a 12-s simulation window, with burst stimuli delivered during the 5–12 s interval. Within this period, bursts were administered at frequencies ranging from 1 to 8 Hz. Simulations were conducted under two distinct network states ‘awake’ (Fig. S4) and ‘sleep’ (Fig. 8), which were differentiated by changes in intrinsic and synaptic parameters to reflect state-dependent dynamics.

## 3. Results

### 3.1. Mthal stimulation modulates wide motor cortical area

**Fig. 2.**
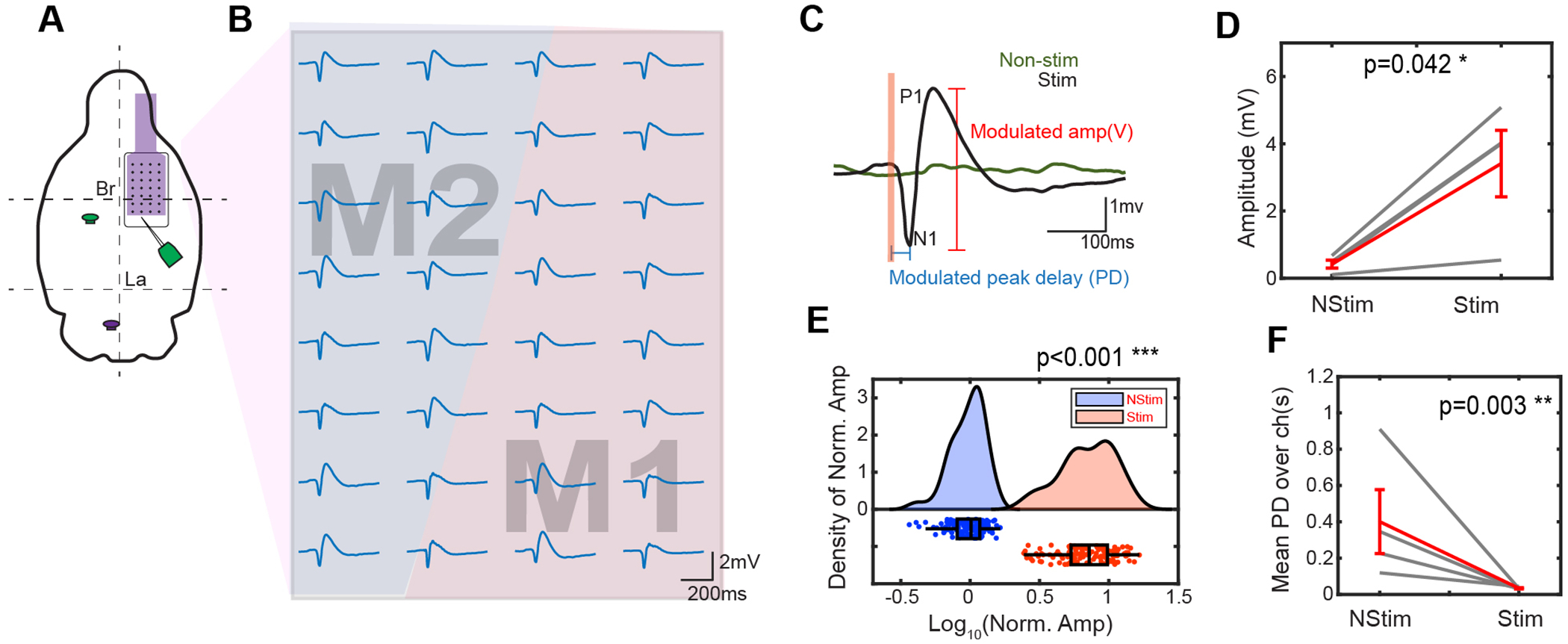
Large-area cortical modulation by Mthal stimulation. (A) Experimental setup for electrocorticography (ECoG) recordings and Mthal stimulation (Stim). Recording and reference/ground screws are shown in purple; stimulation and ground screws are shown in green. (B) Trial-averaged ECoG waveforms across cortical channels during Mthal stimulation (blue) of an example rat. M1 and M2 regions are indicated. Scale bars: 200 ms (horizontal), 2 mV (vertical). Burst stimulation with frequency = 1Hz, PW = 1 ms, 5 biphasic pulses (k = 5) were applied. (C) Example traces illustrating modulation of the first negative peak (N1), first positive peak (P1), and peak delay (PD) during stimulation (black) versus non-stimulation (NStim, dark green). Scale bar: 100 ms, 1 mV. (D) N1 amplitudes across animals (n = 4) in Stim versus NStim conditions. Each gray line represents an individual animal; red line indicates group mean ± standard error of the mean (SEM). *p = 0.042, paired two-tailed *t*-test. (E) Normalized peak amplitudes (Stim or NStim divided by mean NStim) across all channels from four animals. Density scatter plot with overlaid box plot shows median, interquartile range (for 25th, 75th percentiles), and extended whiskers (for 5th, 95th percentile). ***p < 0.001. (F) Mean peak delay (PD) across animals (n = 4). Gray lines represent individual animals; red line shows group mean ± SEM. **p = 0.003, paired two-tailed *t*-test.

Motor thalamus projects to distributed motor cortical regions including M1 and M2 cortex [19,33]. To assess the spatial extent of Mthal stimulation effects, we recorded cortical activity using an ECoG array covering M1 and M2 during stimulation (Fig. 1A middle panel, Fig. 2A). Mthal stimulation elicited clear N1 and P1 peaks across all 32 ECoG channels (Fig. 2B–C, Fig. S2). Stimulation significantly modulated the N1 peak amplitude (paired two-tailed *t*-test, *p* = 0.042*; Fig. 2D). Amplitude distributions between stimulation and non-stimulation conditions were significantly different across channels (***p = 1.07 × 10^−12^; Fig. 2E). The N1 peak latency was 33 ms following stimulation (**p = 0.003; Fig. 2F). These findings confirm that Mthal stimulation modulates widespread cortical activity spanning both primary and secondary motor cortices.

### 3.2. Scalability of cortical activity by Mthal stimulation parameters

To explore how Mthal stimulation parameters influence cortical activity, we systematically varied burst stimulation parameters (Fig. 1B). Effective stimulation requires balancing charge while maximizing modulation effects [34,35]. Pulse amplitude, pulse width, and number of pulses are all capable of impacting stimulation efficacy. We modulated the pulse width and the number of pulses during burst stimulation to achieve varying levels of stimulation, as evidenced by intracortical LFP recordings from layers 3–5 of M1/M2 (Fig. 1A, right panel).

**Fig. 3.**
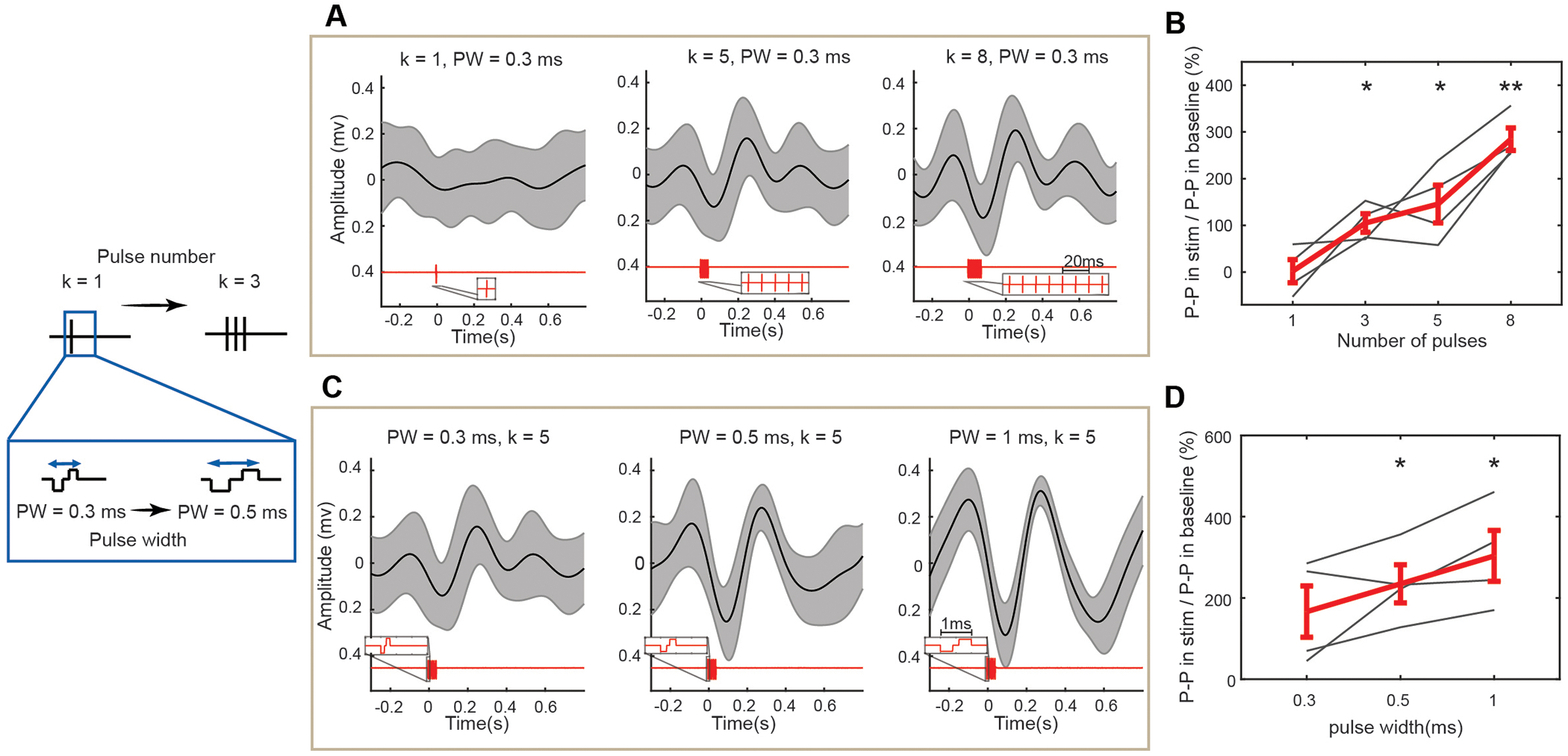
Burst stimulation parameters for scalable cortical excitation. (A) Trial-averaged LFP responses following Mthal stimulation with 1, 5, or 8 pulses (PW = 0.3 ms). Black lines indicate mean amplitude across trials; shaded regions denote ± SEM. Red trace indicates the timing and structure of the burst stimulation waveform. (B) Peak to peak (P–P) amplitude normalized to baseline increases with pulse number with k = 1 to 8 (n = 4). Gray lines represent individual animals; red line indicates group mean ± SEM. *p < 0.05, **p < 0.01; paired two-tailed *t*-test. Non-significant differences are not shown. (C) LFP responses for different PWs (0.3, 0.5, and 1 ms) at fixed pulse number (k = 5). Shown using the same layout as in (A). (D) P-P amplitude increases significantly with PW (n = 4). Gray lines represent individual animals; red line indicates group mean ± SEM. *p < 0.05; paired two-tailed *t*-test. Non-significant differences are not shown.

Modulated activity increased as the number of pulses in burst stimulation was increased. Burst stimulation with 5 and 8 pulses induced evoked activity, whereas single biphasic stimulation did not produce significant modulation (Fig. 3A). The peak-to-peak (P-P) amplitude of the LFP response remained unchanged following single-pulse stimulation. Increasing the number of pulses resulted in a progressive increase in peak-to-peak amplitude of the evoked LFP signals (Fig. 3B). Larger pulse widths providing larger charge yielded greater peak-to-peak LFP amplitudes (Fig. 3C and D). Both pulse width and pulse number increased cortical activity, suggesting that charge level in Mthal stimulation can be fine-tuned to achieve desired cortical modulation levels.

### 3.3. Mthal stimulation promotes motor cortical co-firing activity

**Fig. 4.**
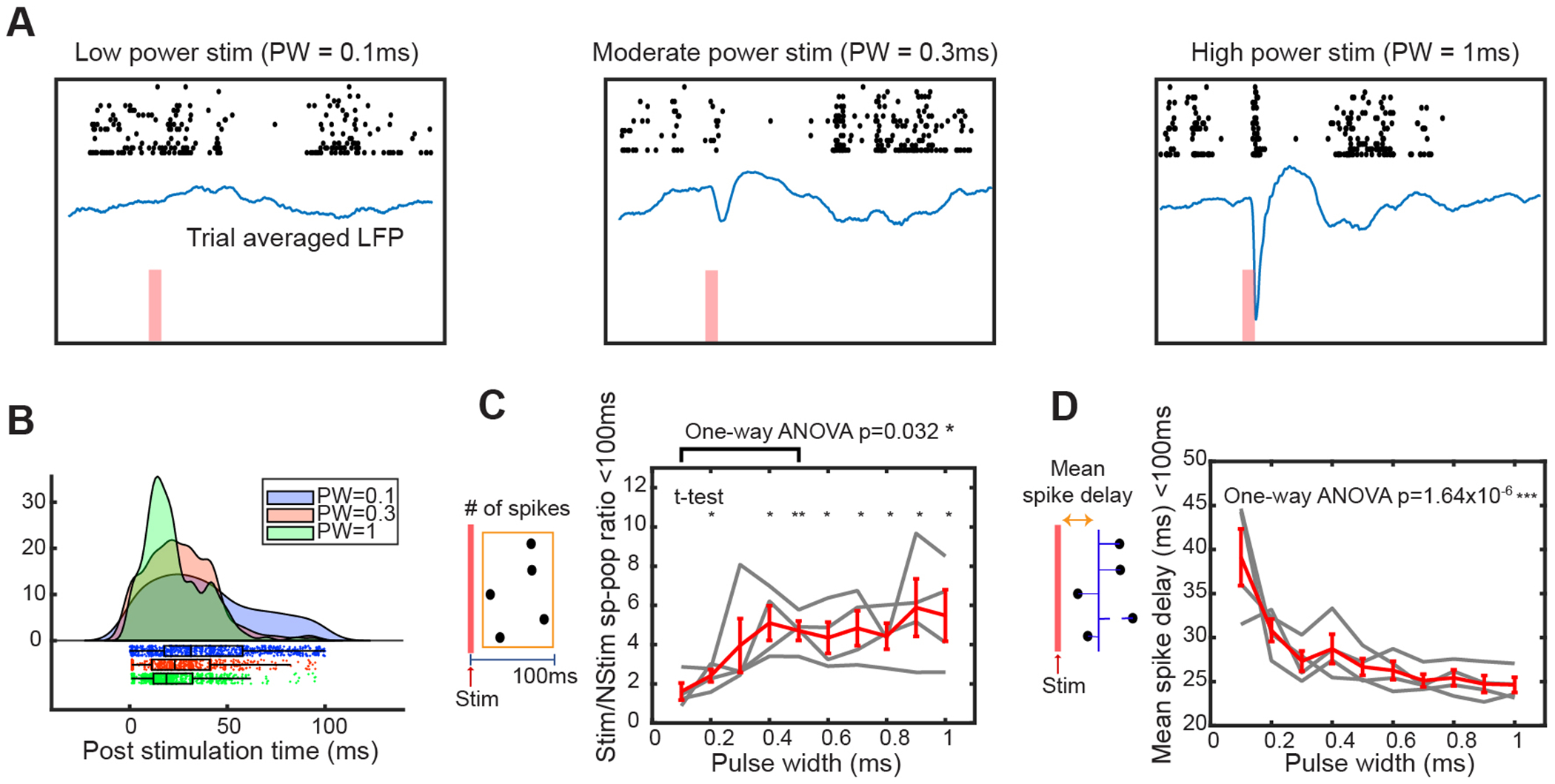
Population response to Mthal stimulation with varying charge densities. (A) Cortical spike raster plots and trial-averaged LFPs (blue) aligned to stimulation onset (red box) under low (PW = 0.1 ms), moderate (PW = 0.3 ms), and high (PW = 1 ms) charge density conditions. The raster plot on the top shows that each horizontal row represents a single unit, while each black dot indicates the occurrence of a spike at a specific time. Raster plot shows 14 units (subset of recorded units from one session). (B) Temporal distribution of spike events for each PW (color-coded: 0.1 ms, green; 0.3 ms, red; 1 ms, blue). Box plots represent spike timing with median, interquartile range, and 95 % confidence notches. 1000 spikes were randomly sampled per condition. (C) Ratio of evoked spike counts (Stim/NStim) within 100 ms post-stimulation increases significantly from low to moderate PW. Gray lines represent individual animals and red line shows group mean ± SEM (n = 4). One-way ANOVA, *p = 0.032. Unpaired two-tailed *t*-test for post hoc comparisons: *p < 0.05, **p < 0.01, and non-significant differences are not shown. (D) Mean spike delay within 100 ms post-stimulation decreases with increasing PW (n = 4). One-way ANOVA, ***p = 1.64 × 10^−6^.

To investigate the effects of Mthal stimulation on cell firing in motor cortex, we performed electrophysiological recordings in M1 following electrical stimulation in Mthal (Fig. 1A right panel). We found that neuronal firing patterns differed across stimulation intensities. High charge stimulation (5 burst pulses with PW = 1 ms) induced stronger, more synchronized co-firing, whereas low and moderate charge densities (5 burst pulses with PW = 0.1–0.3 ms) produced more dispersed firing (Fig. 4A). As stimulation intensity increased, more cortical neurons exhibited stimulus-locked spiking, while Mthal stimulation with 0.1 ms pulse width (with 5 pulses) induced broader, upmodulated activity over a wider time window (Fig. 4B). The total spike count increased with stimulation intensity, but cortical spiking plateaued beyond a pulse width of 0.4 ms (one-way ANOVA, *p = 0.032; Fig. 4C). Spike timing became more precise with larger pulse width stimulation, converging around 30 ms post-stimulation (***p = 1.64 × 10^−6^; Fig. 4D).

In Fig. 3, cortical activation patterns in LFP increased proportionally with stimulation intensity (i.e., stimulation intensity by burst pulse number and pulse width). However, the evoked spike response within 100 ms after stimulation showed a different trend. While spike firing increased proportionally under low-to-moderate Mthal stimulation intensities (5 pulses with PW = 0.1–0.3 ms), this relationship did not persist at high intensities (5 pulses with PW ≥ 0.4 ms). In this higher range, spike firing no longer scaled with stimulation intensity. The firing pattern differences at moderate-to-high stimulation intensities (5 pulses with PW = 0.3–1 ms) were evident in the spike time-delay distributions. Although both conditions engaged a similar range of spike firing populations, moderate-intensity stimulation (5 pulses with PW = 0.3 ms) produced a broader firing distribution, whereas high-intensity stimulation (5 pulses with PW = 1 ms) resulted in a sharper and more temporally confined firing pattern (Fig. 4B). These findings suggest that low-intensity Mthal stimulation (5 pulses with PW < 0.3 ms) primarily modulates cortical network excitability over a broad temporal window, whereas high-intensity stimulation (5 pulses with PW ≥ 0.4 ms) engages stronger thalamocortical coupling, producing a brief, synchronized population [36].

### 3.4. Post silencing and rebound after Mthal stimulation

**Fig. 5.**
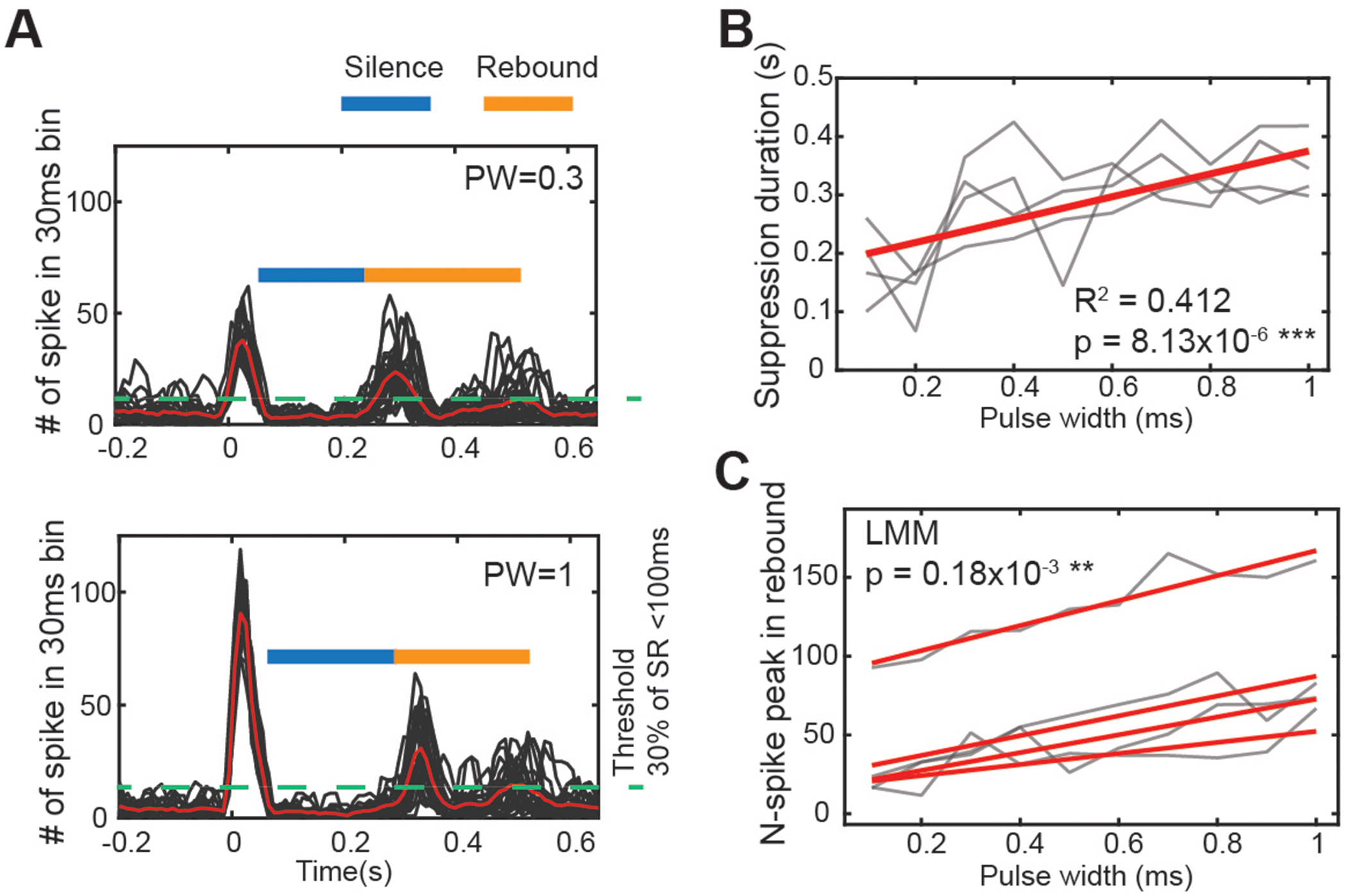
Post-stimulation suppression and rebound. (A) Binned spike rates (30 ms bins) following Mthal stimulation at PW = 0.3 ms (top) and 1 ms (bottom). Individual trials are shown in black; trial average is in red. Suppression (blue) and rebound (orange) periods were defined based on a threshold of 30 % below baseline firing rate within the first 100 ms post-stimulation (green dashed line). (B) Suppression duration for each animals (gray lines) and group linear fit (red). Suppression duration increases linearly with PW. Linear regression: R2 = 0.412, ***p = 8.13 × 10-6. (C) Peak rebound spike rates for each animals (gray) and linear fit (red), showing a significant increase with PW. Linear mixed model: **p = 0.0018.

Following the initial augmentation of cortical firing rates (Fig. 4, <100 ms post stimulation), Mthal stimulation induced two additional phases of modulation: a longer-lasting suppression phase followed by rebound excitation. Using a 600 ms post-stimulation window, we observed that high-intensity stimulation caused strong initial co-firing but was followed by a prolonged inhibitory phase before a rebound response (Fig. 5A). The converged spike population and averaged spike delay in moderate-to-high Mthal stimulation (5 pulses with PW = 0.3–1 ms) was affected by the silencing after the initial strong co-firing in Fig. 4. The duration of this suppression increased with stimulation intensity (Fig. 5B). Following this phase, cortical rebound firing emerged, likely driven by TC neuron excitation through LVA calcium (T-type) channels [37]. High stimulation intensity (5 pulses with PW ≥ 0.4 ms) resulted in stronger rebound responses with larger number of spikes in rebound duration (Fig. 5C), suggesting that stimulation-induced inhibition and rebound effects play a key role in shaping cortical excitability states.

### 3.5. Low-frequency resonance emerges from dynamic state transitions

Next, we sought to characterize how ongoing stimulation with burst stimulation pulses would interact with stimulation-induced and intrinsic cortical dynamics. Based on our single-burst stimulation data (Fig. 5), we hypothesized that stimulation of Mthal at 8Hz would yield diminishing efficacy to drive cortical modulation, as stimulation bursts would arrive during the depressed phase of the prior stimulation. In contrast, we hypothesized that stimulation at 3–4 Hz would yield persistent or facilitating efficacy, since burst stimulation would arrive during the rebound phase of the prior stimulation.

**Fig. 6.**
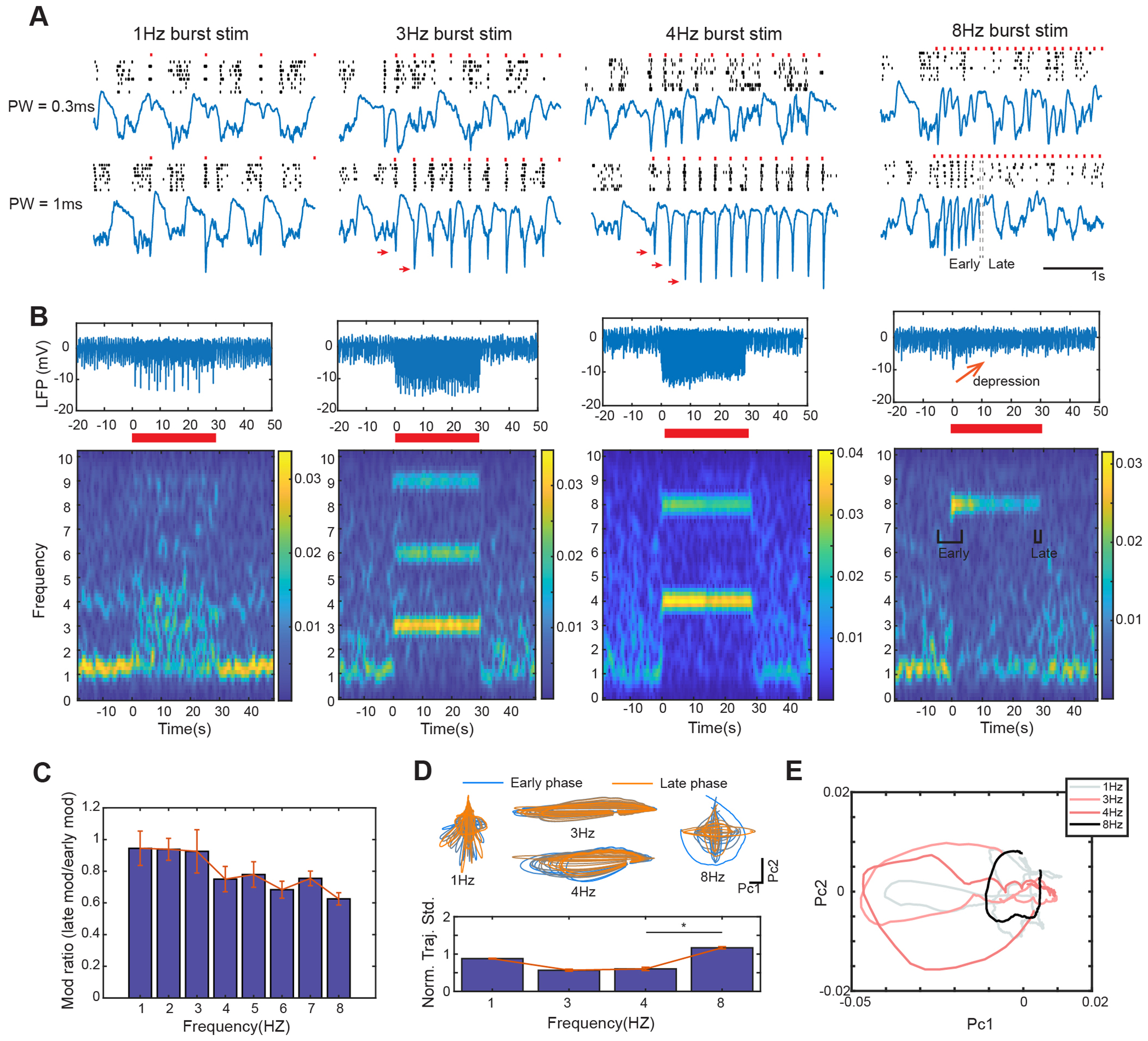
Dynamic state causes low-frequency stimulation preference. (A) Cortical responses during continuous Mthal stimulation at 1, 3, 4, and 8Hz with 5 burst pulses. Single example trials are shown for each stimulation condition. Raster plots (each row representing a unit) and corresponding LFP traces shown for PW = 0.3 ms (top) and 1 ms (bottom). Red dots indicate stimulation onset; red arrows highlight progressive increases in LFP upstate during stimulation. (B) Representative LFP traces (top) and spectrograms (bottom) for each frequency condition over a 70s window (PW = 1 ms). The 30s stimulation period is marked by a red bar. Stimulation at 3–4Hz shows sustained activity, whereas 8Hz induces progressive signal depression (orange arrow). (C) LFP modulation ratio (late/early P–P amplitude) as a function of stimulation frequency. Bars indicate group mean ± SEM across animals (n = 4). (D) (top) PCA trajectories and (bottom) normalized trial-to-trial trajectory standard deviation of LFP signals during early (blue) and late (orange) stimulation phases. Errorbar shows group mean ± SEM. Paired two-tailed *t*-test: *p < 0.05, and non-significant differences are not shown. (E) Trial-averaged PCA trajectories with Mthal stimulation frequencies (1, 3, 4, and 8Hz), revealing more stable and separable dynamics at 3–4Hz compared to 1 or 8Hz.

Indeed, we found that at 3–4 Hz, stimulation enhanced co-firing and LFP amplitude during early stimulation phases, whereas 8 Hz stimulation progressively weakened cortical responses (Fig. 6A–B). The late phase of 8 Hz stimulation (>20 s) showed diminished spiking and slower phase-locked activity, indicating a depressed cortical state (Fig. 6A, right panel). During 30s of continuous stimulation, cortical activity of 1–4Hz Mthal stimulation is relatively stationary compared to faster than 4Hz stimulation (Fig. 6B–C). When stimulating at 3–4 Hz, major frequency components in motor cortex mirrored that of input stimulation frequency in Mthal. However, 1Hz and 8Hz of stimulation led to broader low-frequency band modulation and a non-stationary depressed effect, respectively (Fig. 6B).

PCA of cortical LFPs revealed that 3–4 Hz stimulation produced the most stable showing small trajectory standard deviation and repeated cortical responses (Fig. 6D), whereas 1 Hz and 8 Hz stimulation resulted in less reliable and more depressed activity showing decreased LFP modulation in late phase for 8Hz (Fig. 6C). Neural trajectory analysis further confirmed that 3–4 Hz stimulation optimized cortical excitability, while slower or faster frequencies produced weaker or noisier cortical dynamics (Fig. 6E). Taken together, these results suggest that the cortical state induced by single-burst burst stimuli governs the most effective parameters for ongoing stimulation.

### 3.6. Frequency dependent spike co-firing activity

**Fig. 7.**
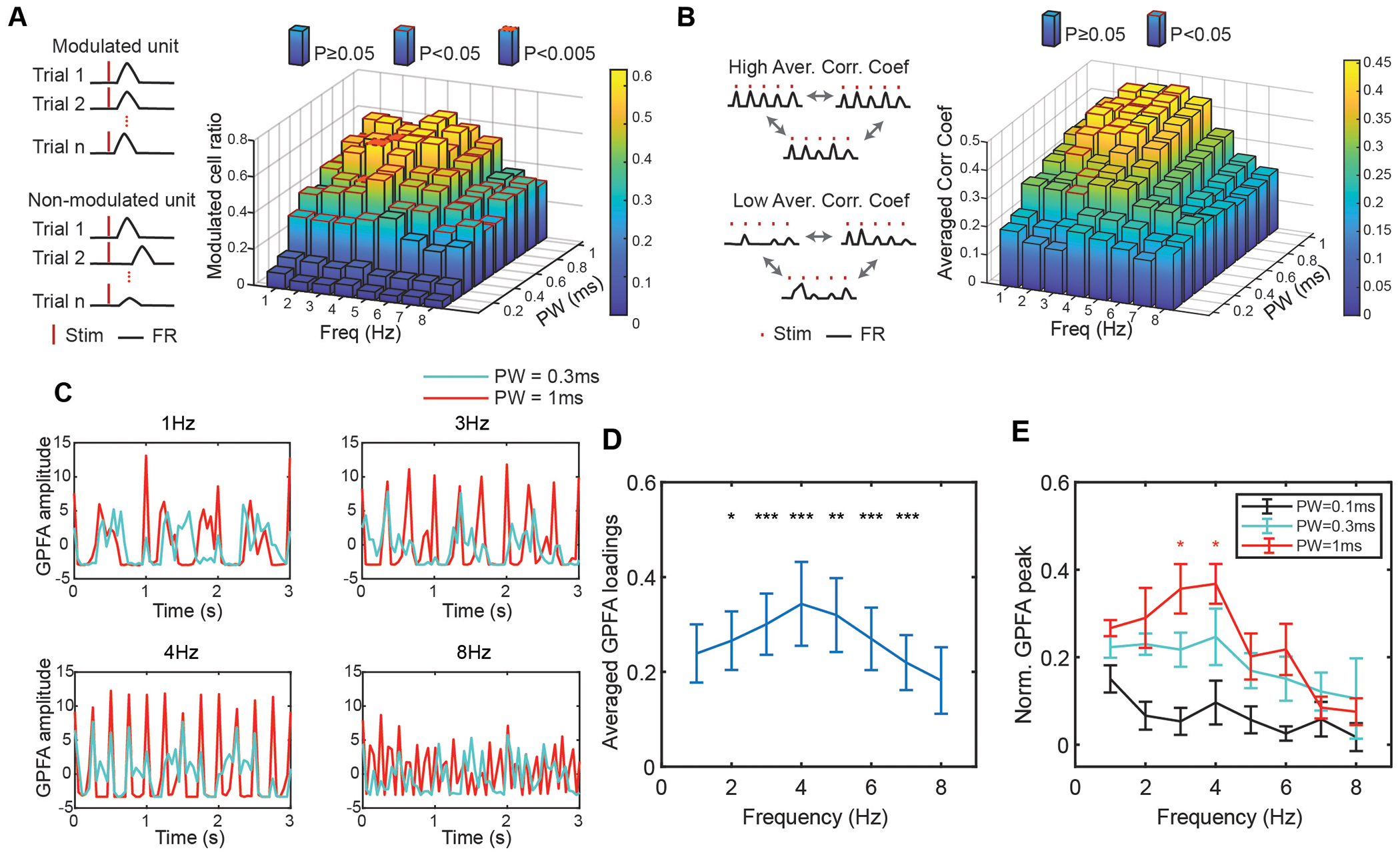
Frequency-dependent spike co-firing activity. (A) (Left) Examples of modulated and non-modulated cells. (Right) Ratio of significantly modulated cortical cells (Stim vs. baseline) across stimulation frequencies (1–8 Hz) and PWs (0.1–1 ms), visualized in a 3D bar plot. (B) (Left) Schematic of high versus low spike-time correlation. (Right) Average pairwise correlation coefficients among all cell pairs at each frequency and PW condition, shown in 3D bar plot. (C) GPFA amplitude traces during Mthal stimulation at 1, 3, 4, and 8 Hz. GPFA results shown for PW = 0.3 ms (cyan) and 1 ms (red). (D) Mean GPFA loadings across stimulation frequencies (1–8 Hz), averaged across animals (n = 4). Bars indicate SEM. Paired one-tailed *t*-test vs. NStim: *p < 0.05, **p < 0.01, ***p < 0.001. (E) Normalized GPFA peak amplitude across stimulation frequencies and PWs (0.1, 0.3, 1 ms). Asterisks indicate significant increases compared to NStim (paired one-tailed *t*-test, n = 4).

We first analyzed cell activity modulated by Mthal stimulation. Cells that showed significant entrainment to Mthal stimulation (Fig. 7A left panel) were identified and used to calculate the proportion of modulated cells. A significant increase in the proportion of modulated cells was observed when transitioning from low to moderate stimulation intensity (5 burst pulses with PW = 0.1–0.3 ms, Fig. 7A right panel). Mthal stimulation frequencies faster than 6 Hz did not further increase the number of modulated cells from moderate to high stimulation intensities, whereas frequencies below 5 Hz continued to show an increase in the modulated cell ratio with rising stimulation intensity. Notably, stimulation around 4 Hz appeared to be the most effective frequency range for enhancing cell modulation at moderate to high intensities (5 pulses with PW = 0.3–1 ms, Fig. 7A and Fig. S3A and B).

The cell activity coupling results demonstrated that correlation coefficients increased with rising stimulation intensity. However, stimulation frequencies faster than 6 Hz did not show a clear increase in correlation with further increases in stimulation intensity (Fig. 7B and Fig. S3C and D). These findings suggest that Mthal stimulation below 5Hz entrained more cells, and their activities were more strongly coupled.

We further analyzed global co-firing activity using GPFA [38]. Compared to the GPFA results at 8 Hz, Mthal stimulation at 1–4 Hz with a 1 ms pulse width elicited stronger co-firing activity than stimulation with a 0.3 ms pulse width (Fig. 7C). GPFA loadings, which reflect the contribution of individual cells to the GPFA factor, were maximal at 4 Hz and declined at higher stimulation frequencies (Fig. 7D). Furthermore, peak GPFA activity following Mthal stimulation was significantly higher than NStim at 3–4 Hz, whereas stimulation frequencies above 5 Hz produced markedly lower peak activities (Fig. 7E).

Interestingly, the optimal frequency (~4 Hz) aligns with the observed thalamocortical rebound delay (200–300 ms, i.e., 3–5 Hz). Conversely, inefficient frequencies (>6 Hz) coincide with the suppression phase (100–200 ms), explaining their weaker modulation effects.

### 3.7. Thalamocortical network modeling reveals importance of ‘rebound’ on stimulation frequency

**Fig. 8.**
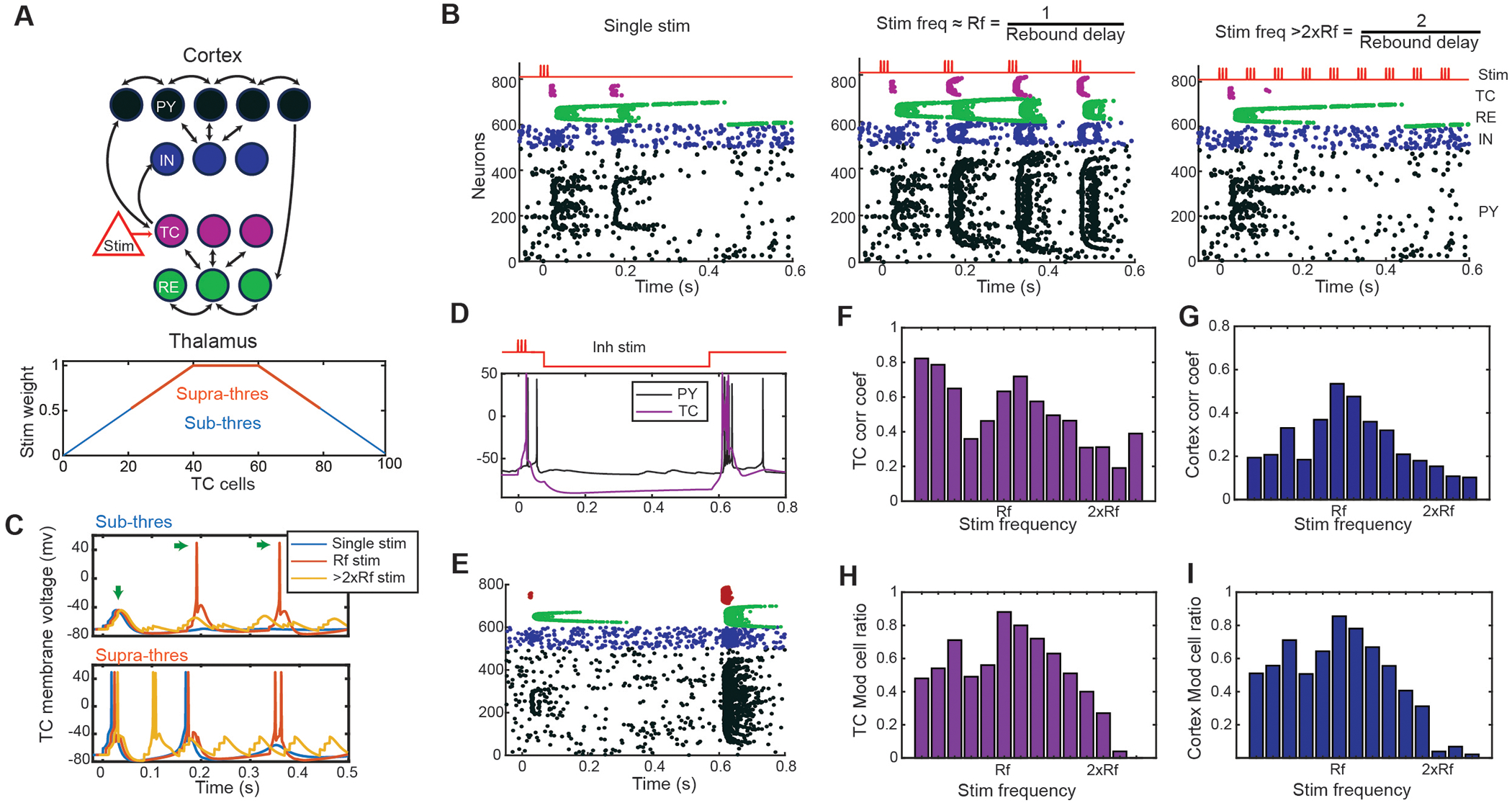
Frequency-dependent effects of Mthal stimulation on the thalamocortical network in silico. (A) Schematic of the TC-Net model (top), including pyramidal (PY), inhibitory (IN), thalamocortical (TC), and reticular (RE) neurons. Stimulation targets TC cells. Bottom: stimulation weights across TC cells with supra-threshold (Orange) and sub-threshold (Blue) distributions. (B) Raster plots showing network spiking under three stimulation conditions: single, resonant-frequency (Rf), and high-frequency (>2 × Rf). Cell types are color-coded: PY (black), IN (blue), TC (purple), RE (green). Red bars indicate burst stimulation. (C) Membrane voltage traces of TC cells across stimulation conditions. Individual examples shown for sub-threshold (top) and supra-threshold (bottom) stimulation. Downward green arrows indicate membrane responses to sub-threshold input, while rightward arrows denote spikes generated by integration of sub-threshold input with rebound excitation. (D) Membrane potentials of PY and TC cells following a hyperpolarizing inhibitory pulse (500 ms). (E) Raster plot showing cortical rebound spiking following release from inhibition in TC cells. (F–G) Spike train correlation coefficients among TC (F) and cortical (G) neurons across stimulation frequencies. (H–I) Proportion of modulated neurons in TC (H) and cortical (I) populations as a function of stimulation frequency.

These empirical observations demonstrated that the effects of Mthal stimulation depend on stimulus parameters and interaction with stimulus-induced cortical dynamics. To investigate potential mechanisms underlying these dynamics, we implemented a TC-Net simulation (see Methods for details). Mthal stimulation was applied to TC cells via non-uniform weighted connections, with 20 % of the cells receiving full weight (1) and the remaining 80 % receiving linearly scaled weights between 0 and 1, delivering burst stimulation (Fig. 8A).

We first examined the effect of a single Mthal stimulation (Fig. 8B, left). Mthal stimulation elicited co-firing in TC cells, subsequently influencing co-firing activity in RE, PY, and IN cells. After the initial co-firing, there was a period of silence, followed by a rebound in TC cell activity (Fig. 8B, left). The rebound activity of the TC cells has been previously well studied across various works [39,40] and arises from the interaction of h and t currents initiated by the suppression of membrane voltage. To confirm this mechanism in our implementation, we applied an additional external hyperpolarizing current (inhibitory stimulation) to TC cells after a burst stimulation. The inhibitory stimulation was applied for a more extended period (500 ms) beyond the spontaneously generated response rebound, which was around 200 ms following a brief stimulation. During this extended period of suppression, the TC cell was in a hyperpolarized state. Upon release from inhibition, the TC cell exhibited a rebound response, leading to a subsequent cortical rebound. (Fig. 8D–E). This delayed the rebound activity while inhibitory input was applied, Confirming, that cortical rebound in this model is driven by TC cell rebound activity.

Next, we investigated the relationship between the Mthal stimulation frequency and the intrinsic rebound frequency (resonant frequency, Rf), specifically comparing stimulation at Rf (3–4 Hz) and at frequencies exceeding twice Rf (over 2 × Rf, ~8 Hz). The initial responses of TC and cortical cells to the first stimulation were similar across Rf and over 2 × Rf conditions. However, significant differences emerged with repeated stimulation. At Rf, a higher proportion of TC and cortical cells became engaged compared to the first stimulation, whereas stimulation at over 2 × Rf resulted in diminished responsiveness and suppression of stimulation effects (Fig. 8B). Stimulation at over 2 × Rf increased the TC membrane potential had a shorter hyperpolarized phase and higher membrane voltage than Rf. Interestingly, this resulted in lack of sustained action potentials in TC cells at 2xRf conditions. In contrast, stimulation at Rf enhanced TC membrane potential when rebound activity was present, allowing for repeated induction of stimulation effects. TC cells that initially received subthreshold stimulation from Mthal input exhibited rebound activity, leading to a greater population of TC cells reaching the action potential threshold with repeated stimulation compared to the first stimulation (Fig. 8C). Consequently, the correlation coefficient of neuronal activity within TC and cortical cells was highest near Rf, indicating a strong synchronization effect (Fig. 8F–G). Furthermore, the number of modulated cells peaked at Rf, whereas stimulation at over 2 × Rf resulted in modulation of only a small subset of TC and cortical cells (Fig. 8H–I). These findings suggest that TC cell hyperpolarization and rebound dynamics play a critical role in shaping the frequency-dependent effects of Mthal stimulation in motor cortical areas.

## 4. Discussion

In this study, we demonstrate that motor thalamus (Mthal) stimulation effectively modulates large-scale motor cortical activity. Our findings indicate that Mthal stimulation can elicit cortical excitation, with the effects being scalable based on stimulation parameters. Furthermore, we identified that low-frequency Mthal stimulation (3–4 Hz) optimally enhances cortical co-firing activity, a phenomenon that we attribute to thalamocortical (TC) cell hyperpolarization and rebound excitation. These results provide new insights into how Mthal stimulation can be leveraged as an efficient method for modulating cortical dynamics by harnessing the intrinsic properties of Mthal activity.

### 4.1. Efficient cortical modulation via Mthal stimulation

Traditional direct cortical stimulation is commonly used to enhance cortical excitability, but its effects are often limited to localized areas due to the exponential decay of the electric field. In contrast, Mthal stimulation indirectly modulates the cortex through widespread thalamocortical projections, enabling broader and more synchronized activation of motor cortical regions (Fig. 2).

Moreover, we demonstrated that burst stimulation parameters, such as pulse number and pulse width, can be precisely tuned to modulate the degree of cortical activation (Fig. 3), providing a flexible and scalable approach to controlling excitability.

Importantly, recent findings [25] showed that thalamic stimulation can dynamically shape cortical trajectories in real time and induce circuit-level plasticity that supports motor learning. Their work suggests that targeting the motor thalamus offers a more effective strategy than direct cortical stimulation for engaging network-level plasticity and facilitating behaviorally relevant learning processes. In this context, Mthal stimulation not only enables spatially extensive and synchronized modulation, but also aligns with mechanisms of adaptive circuit remodeling and motor network reinforcement.

Direct cortical stimulation requires relatively high stimulation intensities to achieve broad cortical activation. In contrast, our study suggests that Mthal stimulation can achieve significant cortical modulation with relatively much lower current charge. By varying pulse width, pulse number, and burst frequency we test the parameter space that can be most effective for network modulation.

### 4.2. Thalamic rebound and frequency-dependent effects of Mthal stimulation

One of the key findings of this study is that Mthal stimulation most effectively modulates cortical excitability at stimulation frequencies of 3–4 Hz. Our results suggest that this effect is mediated by thalamocortical rebound excitation, a mechanism by which TC cells exhibit delayed excitability following hyperpolarization. Specifically, Mthal stimulation at 3–4 Hz aligns with the intrinsic rebound frequency of TC cells (200–300 ms delay, corresponding to 3–5 Hz), allowing for repeated activation cycles that optimize cortical co-firing (Figs. 6–7).

Conversely, stimulation at frequencies above 5 Hz produced smaller cortical spike responses (Fig. 7), likely due to stimulation occurring during the suppression phase of TC activity. At 8 Hz, cortical responses exhibited progressive attenuation, leading to a depressed cortical state over time (Fig. 6). Our computational modeling further supports these findings, demonstrating that stimulation at the optimal frequency (3–4 Hz) enhances cortical synchronization, while higher frequencies disrupt the natural rebound cycle, thereby reducing overall efficacy (Fig. 8).

The Mthal stimulation recordings were obtained under anesthesia, a condition that enhances slow cortical rhythms. These dynamics may be reduced or temporally shifted in the awake state. A study related to cortical stimulation in awake humans and animals has shown that cortically driven thalamic activation can elicit rebound responses at ~150–250 ms [37] which suggests a resonance frequency approximately 1–2 Hz faster than that observed under anesthesia. Thus, because anesthesia strengthens slow oscillations, it may enhance the cortical effect of Mthal stimulation, although the underlying rebound mechanism remains physiologically relevant across brain states (Fig. S4).

### 4.3. Comparison of continuous DBS versus burst modulation

Our burst-modulated approach engages physiological rebound mechanisms within thalamocortical circuits. Notably, 3–4 Hz stimulation significantly enhanced cortical co-firing activity, a temporal pattern known to facilitate Hebbian plasticity. This synchronous activation is likely to strengthen thalamocortical synapses, thereby reinforcing excitatory drive to motor cortex and supporting cortical activity [25] without inducing tremor-like behavioral manifestations, suggesting that burst-modulated Mthal stimulation enhances cortical network activity without causing unintended motor outputs [26]. Such mechanisms are particularly relevant in the context of stroke, where restoring cortical excitability is essential for functional recovery [7,9,41]. Supporting this, recent studies have shown that stimulation of the motor thalamus at frequencies of 50–80 Hz can enhance muscle activity in stroke patients [24], indicating that Mthal stimulation can directly facilitate motor output in impaired systems. Compared to these protocols, our burst-based low-frequency strategy engages thalamocortical circuits via rebound excitation and synchronized co-firing, potentially offering a complementary or more targeted neuromodulation approach.

In the 8 Hz with suprathreshold condition (i.e., PW = 1 ms), stimulation was delivered as 5-pulse bursts at 100 Hz, producing short epochs of high-frequency input. Because these bursts occur twice as often as in lower-frequency conditions such as 4 Hz, the 8 Hz paradigm more closely approximates the cumulative effects of repeated 100 Hz stimulation. Accordingly, the 8 Hz stimulation with a 1 ms pulse width (Fig. 6A) exhibited an initial period of synchronized activation followed by a gradual attenuation of the LFP and spike response in cortex. This pattern resembles observations from human Vim recordings, in which sustained 100–200 Hz stimulation leads to a progressive reduction of thalamic activity [16]. Consistent with our simulation results in Fig. 8C, showing discontinued spike response of Mthal stimulation over 2xRf, several studies have interpreted such suppression as arising from somatic inhibition due to depolarization blockade [15] or from synaptic fatigue [16]. However, other work has shown that axons can remain excitable under high-frequency stimulation (>100 Hz) even when somatic firing is reduced [17], allowing axonal recruitment to continue shaping cortical activity. Thus, to fully understand how the 8 Hz stimulation with 100 Hz bursting utilized in this study and the conventional high-frequency thalamic stimulation (i.e., suppressing tremor [16,42,43]), further mechanistic studies will be required to disentangle the contributions of somatic inhibition, synaptic depression, and axonal excitation.

### 4.4. Implications for motor function modulation

The ability to selectively modulate cortical activity using Mthal stimulation has important implications for neurorehabilitation and neuromodulation therapies. Previous studies have shown that low-frequency cortical oscillations (1–4 Hz) play a crucial role in motor preparation and execution [4,8]. In addition, thalamocortical input can drive cortical activity to enhance motor related plasticity [23,25,44]. Our findings suggest that Mthal stimulation can enhance these oscillations through indirect thalamocortical activation, potentially improving motor function following neurological injury. Given that cortical activity modulation is a key factor in post-stroke recovery [7,9,11], optimized Mthal stimulation may offer a promising therapeutic approach.

## Supplementary Material

Supp_figure2

Supp_figure3

Supp_figure1

Supp_figure4

## Figures and Tables

**Table 1:** Stimulation condition for each animal

	Animal (Rat)	Stim. amp	Stim. type	Stimulation Frequency^a^
ECoG	ECoG-1	20 μA	Cathodic	1 Hz
	ECoG-2	11 μA	Cathodic	1 Hz
	ECoG-3	11 μA	Cathodic	1 Hz
	ECoG-4	10 μA	Cathodic	1 Hz
Intracortical multiarray	Multiarray-1	15 μA	Cathodic	1–8 Hz
	Multiarray-2	15 μA	Cathodic	1–8 Hz
	Multiarray-3	11 μA	Cathodic	8-1 Hz
	Multiarray-4	15 μA	Cathodic	8-1 Hz

a 1–8 Hz and 8–1 Hz indicate the recording sequences in which stimulation frequency was varied from 1 to 8 Hz and from 8 to 1 Hz, respectively.

## Appendix A. Supplementary data

Supplementary data to this article can be found online at https://doi.org/10.1016/j.brs.2025.103018.

## References

[1] Dipietro L, Poizner H, Krebs HI. EEG correlates of submovements. In: 2011 annual international conference of the IEEE engineering in medicine and biology society. IEEE; 2011. 10.1109/IEMBS.2011.6091730 22256056

[2] Ganguly K, Modulation of neural co-firing to enhance network transmission and improve motor function after stroke. Neuron 2022;110(15):2363–85. 35926452 10.1016/j.neuron.2022.06.024 PMC9366919

[3] Grillner S Biological pattern generation: the cellular and computational logic of networks in motion. Neuron 2006;52(5):751–66. 17145498 10.1016/j.neuron.2006.11.008

[4] Hall TM, de Carvalho F, Jackson A. A common structure underlies low-frequency cortical dynamics in movement, sleep, and sedation. Neuron 2014;83(5):1185–99. 25132467 10.1016/j.neuron.2014.07.022 PMC4157580

[5] Kiehn O Locomotor circuits in the mammalian spinal cord. Annu Rev Neurosci 2006;29(1):279–306. 16776587 10.1146/annurev.neuro.29.051605.112910

[6] Roitman AV, Kinematic analysis of manual tracking in monkeys: characterization of movement intermittencies during a circular tracking task. J Neurophysiol 2004;91(2):901–11. 14561685 10.1152/jn.00261.2003

[7] Khanna P, Low-frequency stimulation enhances ensemble co-firing and dexterity after stroke. Cell 2021;184(4):912–930. e20. 33571430 10.1016/j.cell.2021.01.023 PMC7935019

[8] Lemke SM, Emergent modular neural control drives coordinated motor actions. Nat Neurosci 2019;22(7):1122–31. 31133689 10.1038/s41593-019-0407-2 PMC6592763

[9] Ramanathan DS, Low-frequency cortical activity is a neuromodulatory target that tracks recovery after stroke. Nat Med 2018;24(8):1257–67. 29915259 10.1038/s41591-018-0058-y PMC6093781

[10] Okabe N, Theta frequency electromagnetic stimulation enhances functional recovery after stroke. Trans Stroke Res 2025;16(2):194–206. 10.1007/s12975-023-01202-z PMC11976812 37962771

[11] Choi H, Restoration of temporal separability between beta and movement ensemble co-firing with motor recovery. Neuron 2025;113(24):4263–77. 41067228 10.1016/j.neuron.2025.09.013 PMC12614834

[12] Breit S, Schulz JB, Benabid A-L. Deep brain stimulation. Cell Tissue Res 2004;318:275–88. 15322914 10.1007/s00441-004-0936-0

[13] Lozano AM, Deep brain stimulation: current challenges and future directions. Nat Rev Neurol 2019;15(3):148–60. 30683913 10.1038/s41582-018-0128-2 PMC6397644

[14] Herrington TM, Cheng JJ, Eskandar EN. Mechanisms of deep brain stimulation. J Neurophysiol 2016;115(1):19–38. 26510756 10.1152/jn.00281.2015 PMC4760496

[15] Kiss Z, Neuronal response to local electrical stimulation in rat thalamus: physiological implications for mechanisms of deep brain stimulation. Neuroscience 2002;113(1):137–43. 12123692 10.1016/s0306-4522(02)00122-7

[16] Milosevic L, Physiological mechanisms of thalamic ventral intermediate nucleus stimulation for tremor suppression. Brain 2018;141(7):2142–55. 29878147 10.1093/brain/awy139 PMC6022553

[17] McIntyre CC, Cellular effects of deep brain stimulation: model-based analysis of activation and inhibition. J Neurophysiol 2004;94(4):1457–69. 10.1152/jn.00989.2003 14668299

[18] Bopp R, An ultrastructural study of the thalamic input to layer 4 of primary motor and primary somatosensory cortex in the mouse. J Neurosci 2017;37(9):2435–48. 28137974 10.1523/JNEUROSCI.2557-16.2017 PMC6596845

[19] Bosch-Bouju C, Hyland BI, Parr-Brownlie LC. Motor thalamus integration of cortical, cerebellar and basal ganglia information: implications for normal and parkinsonian conditions. Front Comput Neurosci 2013;7:163. 24273509 10.3389/fncom.2013.00163 PMC3822295

[20] Dacre J, A cerebellar-thalamocortical pathway drives behavioral context-dependent movement initiation. Neuron 2021;109(14):2326–38. 34146469 10.1016/j.neuron.2021.05.016 PMC8315304

[21] Kuramoto E, Two types of thalamocortical projections from the motor thalamic nuclei of the rat: a single neuron-tracing study using viral vectors. Cerebr Cortex 2009;19(9):2065–77. 10.1093/cercor/bhn231 19174446

[22] Mandat T, Hurwitz T, Honey C. Hypomania as an adverse effect of subthalamic nucleus stimulation: report of two cases. Acta Neurochir 2006;148(8):895–8. 16763733 10.1007/s00701-006-0795-4

[23] Yu Y, Han F, Wang Q. Exploring phase–amplitude coupling from primary motor cortex-basal ganglia–thalamus network model. Neural Netw 2022;153:130–41. 35717755 10.1016/j.neunet.2022.05.027

[24] Ho JC, Potentiation of cortico-spinal output via targeted electrical stimulation of the motor thalamus. Nat Commun 2024;15(1):8461. 39353911 10.1038/s41467-024-52477-1 PMC11445460

[25] Ramot A, Motor learning refines thalamic influence on motor cortex. Nature 2025:1–10. 10.1038/s41586-025-08962-8 40335698

[26] Sauerbrei BA, Cortical pattern generation during dexterous movement is input-driven. Nature 2020;577(7790):386–91. 31875851 10.1038/s41586-019-1869-9 PMC6962553

[27] Zobeiri M, The hyperpolarization-activated HCN4 channel is important for proper maintenance of oscillatory activity in the thalamocortical system. Cerebr Cortex 2019;29(5):2291–304. 10.1093/cercor/bhz047 PMC6458902 30877792

[28] Murata Y, Colonnese MT. Thalamic inhibitory circuits and network activity development. Brain Res 2019;1706:13–23. 30366019 10.1016/j.brainres.2018.10.024 PMC6363901

[29] Huguenard JR, McCormick DA. Thalamic synchrony and dynamic regulation of global forebrain oscillations. Trends Neurosci 2007;30(7):350–6. 17544519 10.1016/j.tins.2007.05.007

[30] Chaure FJ, Rey HG, Quian Quiroga R. A novel and fully automatic spike-sorting implementation with variable number of features. J Neurophysiol 2018;120(4):1859–71. 29995603 10.1152/jn.00339.2018 PMC6230803

[31] Cowley BR, DataHigh: graphical user interface for visualizing and interacting with high-dimensional neural activity. J Neural Eng 2013;10(6):066012. 24216250 10.1088/1741-2560/10/6/066012 PMC3950756

[32] Krishnan GP, Cellular and neurochemical basis of sleep stages in the thalamocortical network. eLife 2016;5:e18607. 27849520 10.7554/eLife.18607 PMC5111887

[33] Shepherd GM, Yamawaki N. Untangling the cortico-thalamo-cortical loop: cellular pieces of a knotty circuit puzzle. Nat Rev Neurosci 2021;22(7):389–406. 33958775 10.1038/s41583-021-00459-3 PMC9006917

[34] Cogan SF, Hara S, Ludwig KA. The safe delivery of electrical currents and neuromodulation. In: Neuromodulation. Elsevier; 2018. p. 83–94.

[35] Cogan SF, Tissue damage thresholds during therapeutic electrical stimulation. J Neural Eng 2016;13(2):021001. 26792176 10.1088/1741-2560/13/2/021001 PMC5386002

[36] Na J, Kakei S, Shinoda Y. Cerebellar input to corticothalamic neurons in layers V and VI in the motor cortex. Neurosci Res 1997;28(1):77–91. 9179883 10.1016/s0168-0102(97)00031-x

[37] Kumaravelu K, Grill WM. Neural mechanisms of the temporal response of cortical neurons to intracortical microstimulation. Brain Stimul 2024;17(2):365–81. 38492885 10.1016/j.brs.2024.03.012 PMC11090107

[38] Cunningham JP, Yu BM. Dimensionality reduction for large-scale neural recordings. Nat Neurosci 2014;17(11):1500–9. 25151264 10.1038/nn.3776 PMC4433019

[39] Russo S, Thalamic feedback shapes brain responses evoked by cortical stimulation in mice and humans. Nat Commun 2025;16(1):3627. 40240330 10.1038/s41467-025-58717-2 PMC12003640

[40] Grenier F, Timofeev I, Steriade M. Leading role of thalamic over cortical neurons during postinhibitory rebound excitation. Proc Natl Acad Sci 1998;95(23):13929–34. 9811903 10.1073/pnas.95.23.13929 PMC24971

[41] Kim K, Subthreshold electrical stimulation as a low power electrical treatment for stroke rehabilitation. Sci Rep 2021;11(1):14048. 34234199 10.1038/s41598-021-93354-x PMC8263745

[42] Earhart GM, Effects of thalamic stimulation frequency on intention and postural tremor. Exp Neurol 2007;208(2):257–63. 17920589 10.1016/j.expneurol.2007.08.014 PMC2203380

[43] Lyons KE, Pahwa R. Deep brain stimulation and tremor. Neurotherapeutics 2008;5(2):331–8. 18394574 10.1016/j.nurt.2008.01.004 PMC5084174

[44] Athalye VR, Carmena JM, Costa RM. Neural reinforcement: re-entering and refining neural dynamics leading to desirable outcomes. Curr Opin Neurobiol 2020;60:145–54. 31877493 10.1016/j.conb.2019.11.023

