# Supplementary figures and images for: Effects of low-frequency burst stimulation of the motor thalamus on cortical neural co-firing
Source: Brain Stimul. Author manuscript; Available in PMC 2026 Jul 10. (PMC13353076; doi:10.1016/j.brs.2025.103018)

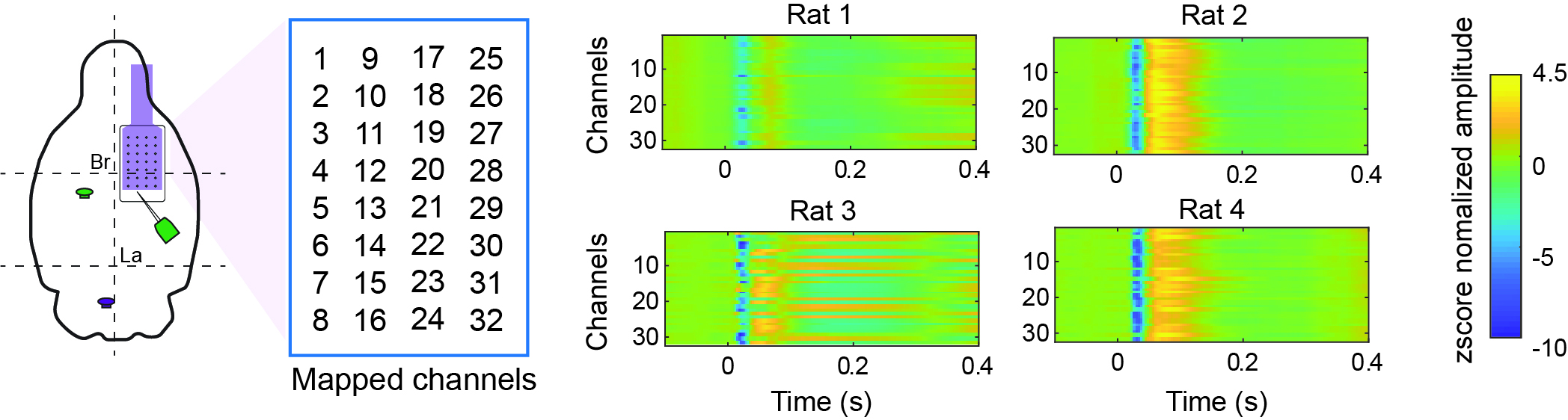

Supplement: Supp_figure2 [file NIHMS2188323-supplement-Supp_figure2.jpg]

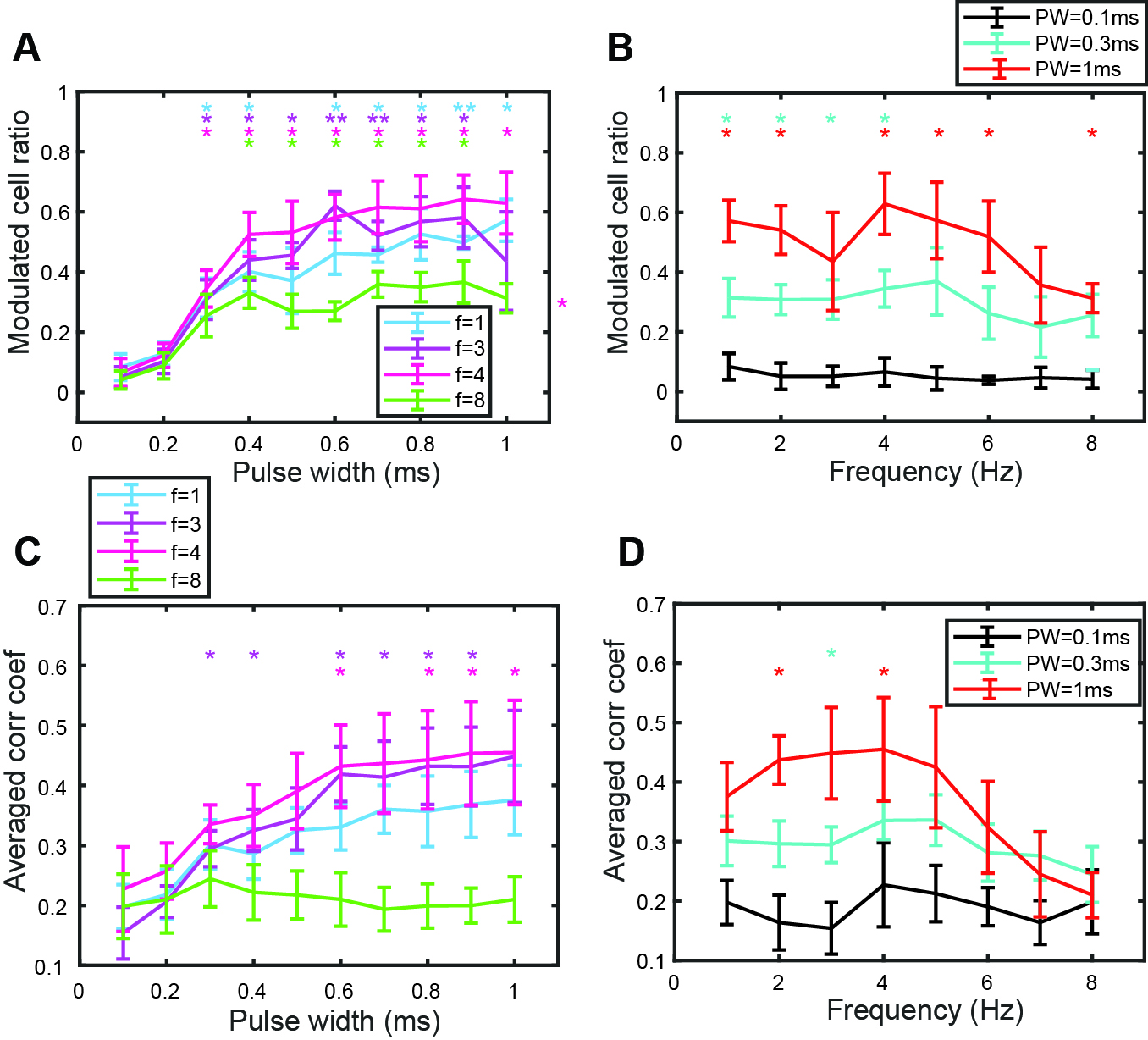

Supplement: Supp_figure3 [file NIHMS2188323-supplement-Supp_figure3.jpg]

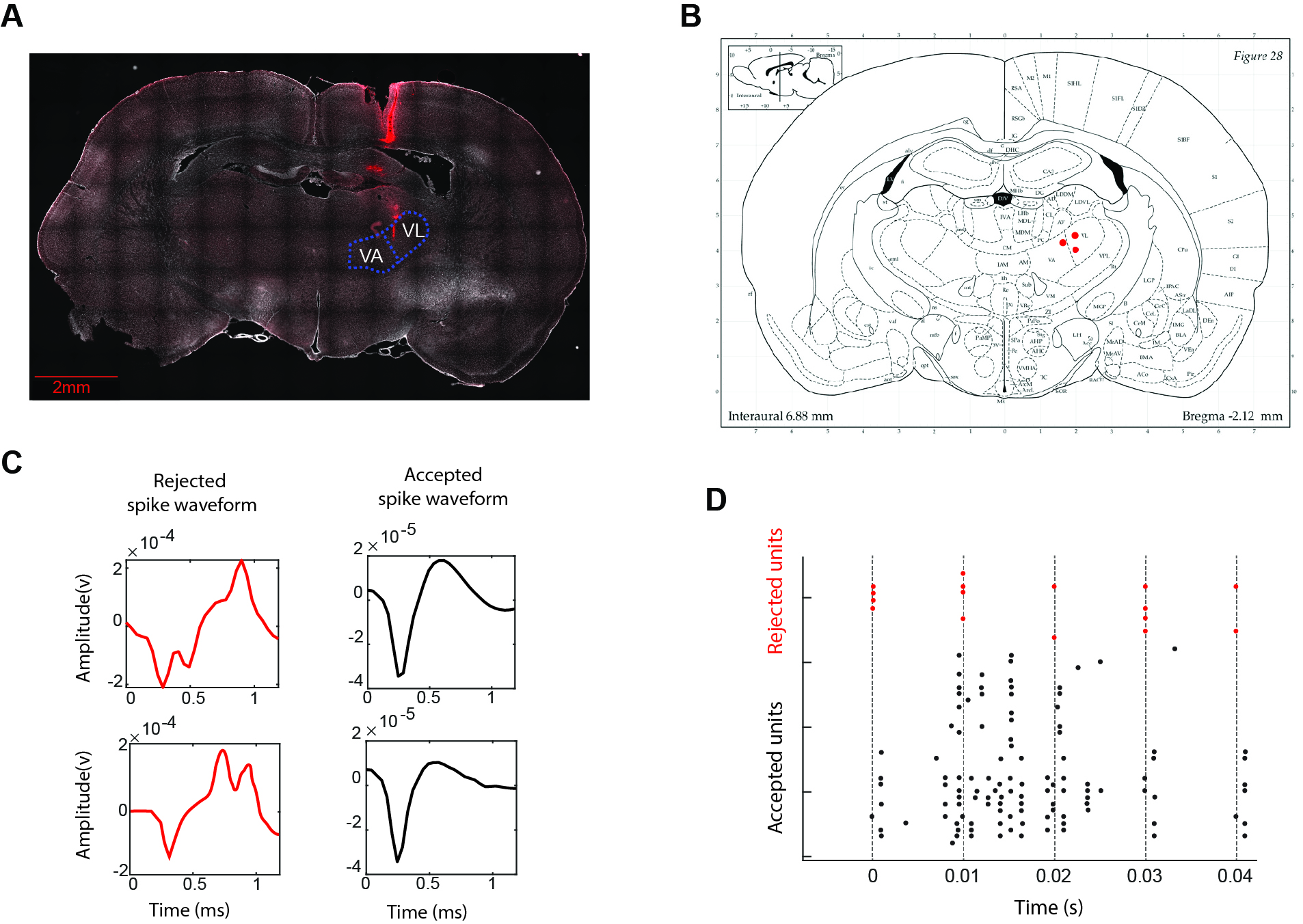

Supplement: Supp_figure1 [file NIHMS2188323-supplement-Supp_figure1.jpg]

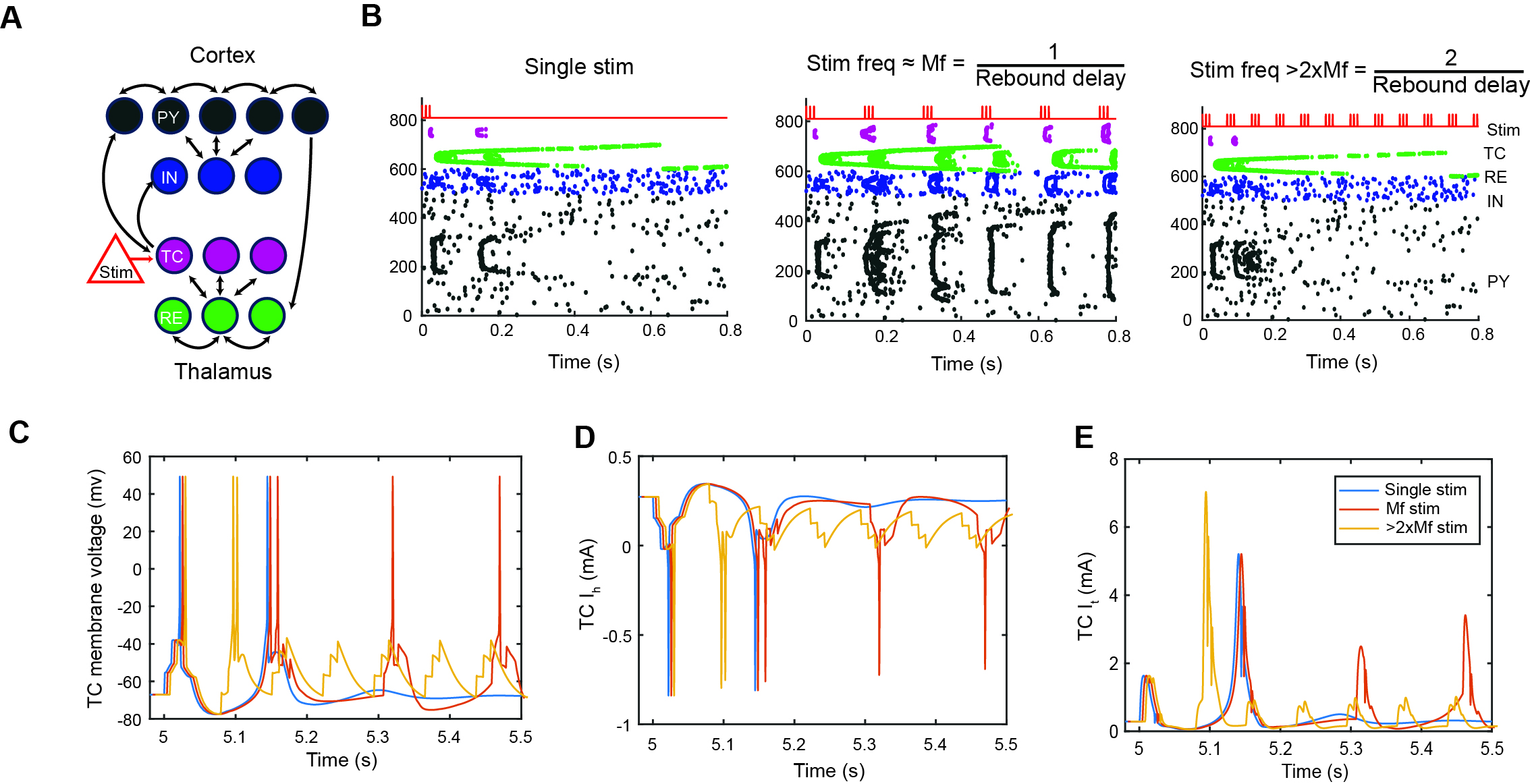

Supplement: Supp_figure4 [file NIHMS2188323-supplement-Supp_figure4.jpg]
